# Misidentification of preformed anti-HLA-DP antibodies leads to antibody-mediated kidney transplant rejection: a case report

**DOI:** 10.1186/s12882-022-02807-6

**Published:** 2022-05-17

**Authors:** Duangtawan Thammanichanond, Chutima Tammakorn, Atiporn Ingsathit, Suchin Worawichawong, Premsant Sangkum

**Affiliations:** 1 Histocompatibility and Immunogenetics Laboratory, Department of Pathology, Faculty of Medicine Ramathibodi Hospital, Mahidol University, Rama VI Road, Bangkok, 10400 Thailand; 2 Division of Nephrology, Department of Medicine, Faculty of Medicine Ramathibodi Hospital, Mahidol University, Bangkok, Thailand; 3 Immunopathology Laboratory, Department of Pathology, Faculty of Medicine Ramathibodi Hospital, Mahidol University, Bangkok, Thailand; 4 Division of Urology, Department of Surgery, Faculty of Medicine Ramathibodi Hospital, Mahidol University, Bangkok, Thailand

**Keywords:** HLA-DP, Donor-specific antibody, Antibody-mediated rejection, Kidney transplantation, Case report

## Abstract

**Background:**

Patients who are HLA-sensitized are at high risk for early antibody-mediated rejection (AMR) and worse outcomes. Therefore, it is crucial to detect the presence of donor-specific antibodies (DSAs) using pretransplant antibody identification and crossmatch assays. An error in antibody identification can lead to disastrous clinical outcomes. We present a case of acute AMR associated with preformed HLA-DPα and HLA-DPβ DSAs that were not identified before transplantation.

**Case presentation:**

A 27-year-old woman received a second kidney transplant from a deceased donor. Her pretransplant panel-reactive antibody level was 94%. The complement-dependent cytotoxicity crossmatch was negative for T and B cells at the time of transplantation. She experienced early acute AMR proven by a kidney biopsy. Single antigen bead testing of the patient’s serum at the time of rejection as well as the pre-second transplant serum revealed strong antibodies against the DPA1*01:03 and DPB1*02:01 alleles in the second donor. These antibodies were not identified by phenotypic bead assay during the patient’s time on the waiting list. The patient was treated with plasmapheresis and anti-thymocyte globulin. However, she experienced abdominal pain on day 37 post-transplantation. Surgical exploration revealed a laceration on the transplanted kidney, which was then repaired. Subsequently, infected hematoma was suspected and the transplanted kidney was removed.

**Conclusion:**

The present case highlights the clinical significance of preformed HLA-DPα and HLA-DPβ DSAs. Accuracy in determination of HLA antibodies before transplantattion is critical for transplant outcome. HLA-DP typing and single antigen bead testing are recommended for a precise antibody interpretation, especially in highly sensitized patients. Careful interpretation of antibody testing results is essential for the success of organ transplantation.

## Background

Human leukocyte antigen (HLA) sensitization is a major barrier to successful kidney transplantation. HLA sensitization typically occurs after immunological challenge by non-self HLA antigens, such as previous organ transplantation, blood transfusions, and pregnancies. Sensitized transplant candidates are at increased risk of rejection and graft loss [1, 2].

With the advent of multiplexed fluorescence-based solid-phase assays (Luminex assays), transplant laboratories have highly sensitive tools for the detection of HLA antibodies. HLA antibody identification using the Luminex platform can be performed on two different types of panels: phenotype panels and single antigen bead (SAB) panels. Phenotype panels have individual beads coated with HLA molecules from a cell line derived from a single individual, which are sometimes called panel-reactive antibody (PRA) beads. Meanwhile, SAB panels have individual beads coated with a single HLA molecule, and allow precise determination of HLA antibody specificities in highly sensitized patients. Therefore, SAB assays are recommended for the detection of pretransplant HLA antibodies in solid organ transplant recipients in The Transplantation Society Consensus Guideline [3]. Nevertheless, HLA laboratories in low-income or middle-income countries do not use SAB testing for waitlisted patients due to insufficient financial resources. Consequently, antibody identifications in these countries are commonly performed by using phenotype panels, which have lower sensitivity and accuracy.

Each HLA-DP molecule is composed of two chains, DPα and DPβ, encoded by DPA1 and DPB1 loci respectively. The DPA1 and DPB1 genes are highly polymorphic. Indeed, 216 DPA1 and 1654 DPB1 alleles have been reported (IPD-IMGT/HLA Database release 3.42.0, October 2020; www.ebi.ac.uk/ipd/imgt/hla) [4]. Anti-DP antibodies have been considered less significant for clinical transplantation because of the low expression of HLA-DP on renal endothelial cells [5]. There is very limited literature on the role of anti-DP antibodies in kidney transplantation. We present the case of a kidney transplant recipient who experienced early antibody-mediated rejection (AMR) who had alloantibodies directed against donor-specific DPA1 and DPB1 alleles. The phenotypic bead assay was unable to identify these anti-DPα and anti-DPβ donor-specific antibodies (DSAs) in pretransplant serum, underscoring the inadequacy of the phenotypic bead assay for antibody identification. An analysis of epitope-based antibody reactivity confirmed that the anti-DPα and anti-DPβ DSAs were specific for epitopes shared by the DPA1 and DPB1 alleles in the first and second donors, respectively.

## Case presentation

A 27-year-old woman with end-stage renal disease secondary to chronic glomerulonephritis received a first kidney transplant from a deceased donor in 2014. Recipient and donor HLA typings was carried out at the low-to-medium resolution level for the A, B, DR and DQ loci. The HLA mismatch for the A, B and DR loci was 2-0-1, respectively. HLA-DP typing is not routinely performed in kidney transplant recipients at our center, and thus any DP mismatch between the donor and the recipient was unknown at the time of the transplantation. The pretransplant PRA level measured by the Luminex assay was 0% for both class I and II and the complement-dependent cytotoxicity crossmatch (CDC-XM) was negative. She had not experienced any pregnancies or blood transfusions. At 2 years and 4 months post-transplantation, the grafted kidney function deteriorated. The patient reported immunosuppression non-compliance. The kidney biopsy showed Banff IIA acute T-cell mediated rejection. Subsequently, the patient was placed on dialysis and added to the waiting list for a deceased donor kidney. 2 years later, she received a second kidney transplant from a deceased donor. For the second transplantation, the HLA mismatch for the A, B, DR and DQ loci was 1-1-1-1, respectively. The patient’s class I and II PRA levels were 84 and 94%, respectively. The unacceptable antigens identified by the phenotypic bead assay before the second transplant were A1, A2, A23, A24, A25, A32, A68, A69, A80, B44, B49, B51, B52, B53, B57, B58, B59, B61, B63, B64, B65, DR1, DR4, DR7, DR9, DR10, DR16, DR103, DR53 and DQ4. These unacceptable antigens were not matched with antigens in the second donor and the pretransplant CDC-XM was negative. The kidney transplantation proceeded with cold ischemic time of 17 hours and 23 minutes. The left donor kidney was placed in the left iliac fossa of the recipient. The patient received an anti-CD25 monoclonal antibody as induction therapy. The maintenance immunosuppressive regimen comprised 6 mg/day of tacrolimus (to achieve trough blood levels between 5 and 8 ng/ml), 750 mg b.i.d. of mycophenolate mofetil, and 500 mg of intravenous methylprednisolone on day 1 post-transplant that was gradually tapered to 20 mg/day of oral prednisolone thereafter. The patient required hemodialysis several times due to delayed graft function. At 15 days post-transplantation, she had minimal urine output and her creatinine level was 5.32 mg/dL. An allograft kidney biopsy was performed and revealed active AMR with minimal C4d staining, transplant glomerulitis and peritubular capillaritis (Banff schema: i1, t1, g3, v1, ptc3, ci1, ct1, cg0, cv0, mm0, ah0, c4d1, ti1, iIFTA0). Luminex SAB testing (One Lambda, Canoga Park, CA) of day 17 post-transplant serum showed the presence of anti-HLA class II antibodies, mainly against DP antigens. High-resolution HLA-DP typing of the patient and the second donor was retrospectively performed (Table 1). Analysis of the SAB reactions together with the HLA typing results revealed that the patient had DSAs against DPA1*01:03 (mean fluorescence intensity [MFI]: 22004) and DPB1*02:01 (MFI: 17055). No DSAs against HLA alleles in other loci were detected.

**Table 1:** HLA class I and II typing of the patient and two donors^a^

	A*	B*	C*	DRB1*	DRB3/4/5*	DQB1*	DPA1*	DPB1*
Recipient	11:01	13:01	04:06	12:01	DRB3*01:01	03:01	02:02	02:02
	11:01	40:01	07:02	15:02	DRB5*01:08 N	05:02	–	05:01
First donor	24:02	13:01	03:04	15:02	DRB5*01:01	03:01	**01:03**	**03:01**
	24:03	40:01	03:04	16:02	**–**	05:02	–	**04:01**
Second donor	11:01	13:01	03:04	08:03	DRB3*03:01	03:01	**01:03**	**02:01**
	26:01	38:02	07:02	12:02	**–**	06:01	02:02	02:02

^a^ Bold typeface indicates HLA-DP disparitiesin the donors compared with the recipient

**Fig. 1:**
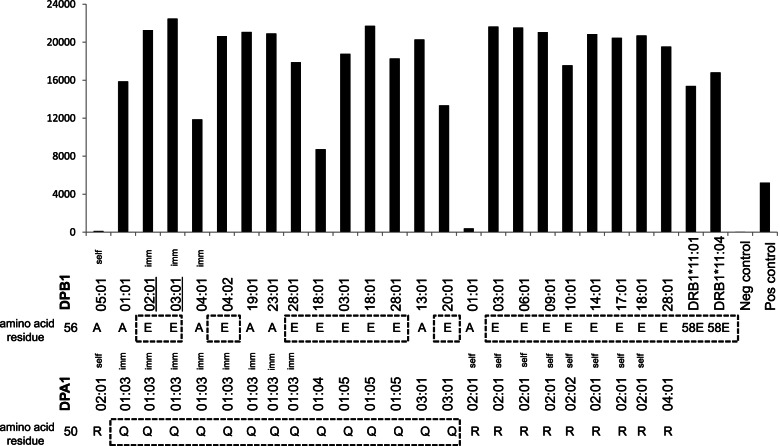
Antibody reactivities in pre-second transplant serum against DP single antigen panel beads. The reactions against the E residue at amino acid position 56 (56E eplet) in DPB1 and against the Q residue at amino acid position 50 (50Q eplet) in DPA1 are shown at the bottom and highlighted in the dashed boxes. DPB1*03:01 in the first donor and DPB1*02:01 in the second donor share the 56E eplet and are underlined. The mean fluorescence intensity cutoff value for a positive result was set at 1000. Beads carrying the patient’s DPA1 or DPB1 alleles are labeled as self, while beads carrying the donors’ DPA1 or DPB1 alleles are indicated as imm (abbreviation for immunizer). Because DPA1*02:01 and DPA1*02:02 have identical allogeneic eplet profiles, DPA1*02:01 is used as a substitute for the patient’s allele. The positive reactions against DR11 alleles are due to cross-reactions with E residue at amino acid position 58 in DR11

To determine whether the detected DSAs were preformed DSAs, we tested the patient’s pre-second transplant serum with the SAB assay. The results revealed that these DSAs were present prior to the second transplantation. High-resolution HLA-DP typing of the first donor revealed that both the first and second donors shared the DPA1*01:03 allele. As the first and second donors did not share any DPB1 alleles, the question arose as to why the patient had antibodies against DPB1*02:01. This question prompted us to investigate whether there were any shared epitopes between the first and second donor DPB1 alleles. An HLAMatchmaker analysis was conducted (HLA-DRDQDP analysis version 3.0; http://www.epitopes.net) [6]. The results showed that the antibodies in the patient’s serum were reactive toward a glutamic acid residue (E) at amino acid position 56 (56E eplet) in DPB1. The 56E eplet was a verified eplet shared by DPB1*03:01 (DPB1 allele in the first donor) and DPB1*02:01 (DPB1 allele in the second donor). In addition, the 56E eplet was shared by several other DPB1 alleles, including DPB1*04:02, *06:01, *09:01, *10:01, *14:01, *17:01, *18:01, *20:01, and *28:01 (Fig. 1). Interestingly, positive reactions against DR11-carrying beads were observed. These reactions arose because DR11 had a polymorphic residue 58E that could cross-react with the 56E eplet on DPB1. Analysis of the patient’s serum reactivity against DPA1 alleles also showed that the antibodies were reactive toward a glutamine residue (Q) at amino acid position 50 (50Q eplet) in DPA1. The 50Q eplet was a verified eplet shared by several DPA1 alleles including DPA1*01:03, *01:04, *01:05, and *03:01. These epitopes shared between different HLA molecules in DPB1 and DPA1 loci can explain the antibody reactions against non-donor-specific DP alleles (Fig. 1).

**Fig. 2:**
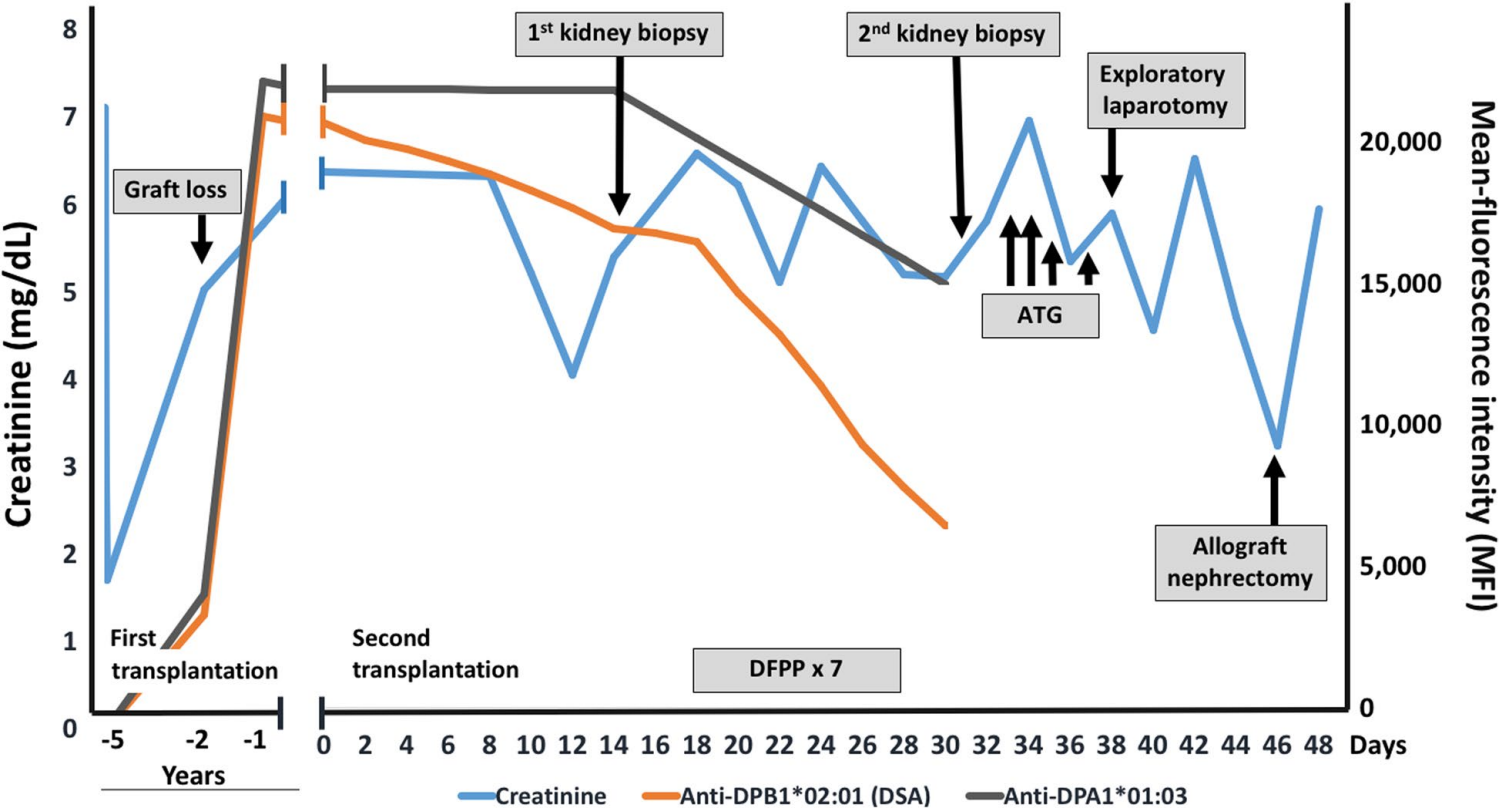
Clinical course of the patient. The first and second kidney biopsies revealed antibody-mediated rejection. The patient was treated with seven sessions of double-filtration plasmapheresis and anti-thymocyte globulin. The patient experienced sudden abdominal pain together with a drop in blood pressure on day 37 post-transplantation. An emergency exploratory laparotomy showed a laceration on the transplanted kidney. After surgical repair, an infected hematoma was suspected and allograft nephrectomy was performed. ATG, anti-thymocyte globulin; DFPP, double-filtration plasmapheresis; MFI, mean fluorescence intensity

The patient was treated with seven sessions of double-filtration plasmapheresis. A subsequent allograft biopsy at 1 month post-transplantation showed persistent active AMR, transplant glomerulitis and peritubular capillaritis (Banff schema: i0, t0, g3, v1, ptc3, ci2, ct2, cg2, cv1, mm0, ah0, c4d0, ti1, iIFTA1). In addition, the MFI for anti-DPA1*01:03 DSAs remained high (MFI: 14126). Her creatinine level was 5.09 mg/dL. The patient was treated with four doses of anti-thymocyte globulin (ATG) at 1-1.5 mg/kg. After the ATG treatment, her urine output increased to 1000-1500 mL/day and her creatinine level decreased to 4.25 mg/dL (Fig. 2). However, the patient experienced sudden left lower quadrant abdominal pain on day 37 post-transplantation (day 7 after the second kidney biopsy). She also had a sudden drop in blood pressure with a significant decrease in hemoglobin level from 8.5 to 4.5 g/dL. An emergency exploratory laparotomy showed a 1-cm laceration at the upper part of the transplanted kidney with arterial spurting. Three to four sites oozing blood from the transplanted kidney and 500 mL of fresh clotted blood were also noted. The laceration was repaired with sutures and the blood clot was removed. She received several blood products including red blood cells, fresh-frozen plasma, and platelets. On the following day, the patient experienced gross hematuria and abdominal extension. Her hemoglobin level decreased from 10.1 to 7.1 g/dL. Ultrasonography showed a suspected pseudoaneurysm at the mid-pole region of the transplanted kidney. Coil embolization was performed for the interlobar branch of the middle pole in the left transplanted renal artery. After the embolization, the patient had fever and abdominal distension. 2 days later, she complained of severe pain at both upper thigh areas. Her antibiotic drug treatment was changed from cefepime to doripenem and colistin. Computed tomography of the whole abdomen showed a large perinephric hematoma. Infected hematoma was suspected. An allograft nephrectomy was performed to remove the source of the infection on day 45 post-transplantation. Old clotted blood at 1500 mL was found at the superior border of the transplanted kidney during the operation. There was also blood oozing from the raw surface and a pseudoaneurysm in the middle pole of the transplanted kidney. After allograft nephrectomy, the fever subsided and the patient remained clinically stable. She was discharged on day 52 post-transplantation. Currently the patient is on regular hemodialysis.

## Discussion and conclusions

Because immunization against foreign HLA antigens in recipients has been recognized as a major barrier to successful solid organ transplantation, determination of unacceptable mismatched HLA antigens has become a routine assessment prior to transplantation. Accurate determination of unacceptable mismatched antigens can prevent early graft rejection and futile organ shipments. Although antibodies against HLA-A, HLA-B, HLA-C, HLA-DR, and HLA-DQ antigens have long been considered unacceptable during virtual crossmatching, anti-DP antibodies are not considered in several countries [7]. The present case demonstrates that pretransplant HLA-DP DSAs are pathogenic and that misidentification of anti-DP antibodies is harmful to organ transplant recipients.

The performance characteristics of solid phase assay for determining HLA antibodies is greatly dependent on the panel composition used in the assay [3]. Because the compositions of target antigens on phenotypic beads are HLA class I or class II proteins from a cell line derived from a single individual, several HLA specificities are present on each bead. The presence of high-frequency antigens on the same beads carrying DP antigens affects the accuracy of antibody identification by phenotypic bead assays [8]. In our case, anti-DP2 antibodies were masked by the presence of anti-DQ7 and anti-DR11 antibodies, which were identified by software analysis of the phenotypic bead assays in serum taken while the patient was on the waiting list for the second transplantation. Retrospectively, SAB testing of the same serum showed that the patient did indeed have anti-DR11 antibodies, although no anti-DQ7 antibodies were detected. Because the DP2 antigen was not listed as an unacceptable antigen and the CDC-XM was negative, this led to acceptance of the offered kidney.

Recent studies have evaluated the association between MFI of HLA-DP antibodies and the results of flow cytometric crossmatch (FCXM) [9, 10]. Simmons et al. analyzed FCXM results in nine patients with HLA-DP antibodies and found that two patients with HLA-DP antibodies with MFI > 8000 had positive crossmatches [10]. On the other hand, FCXM results were negative for 7 patients with MFI of HLA-DP antibodies ranging from 3000 to 8000. A multicenter study in Belgium investigated 20 FCXM using sera with HLA-DPB1 antibodies [9]. Three positive FCXM results were detected. Two sera had HLA-DP DSAs with MFI ranging from 2000 to 4000 and 1 serum with cumulative MFI of 18,676. Seventeen negative crossmatch results were observed in sera with MFI ranging from 1000 to 10,000. A report of two patients with HLA-DPB1 antibodies with MFI > 9000 showed positive FCXM [11]. In contrast, a patient with HLA-DP antibodies with MFI 3000-4000 had negative FCXM [12]. Altogether, these previous reports suggested that HLA-DP DSA MFI > 10,000 could potentially result in positive FCXM. In our case the FCXM was not performed. SAB analysis of pre-second transplant serum revealed that the majority of positive single antigen beads carrying DP alleles had MFI > 10,000. Accordingly, there is a high possibility that FCXM could detect the preformed HLA-DP DSAs in our patient.

The presence of anti-DP antibodies in our patient, who was a re-transplant case, emphasizes the high risk for development of anti-DP antibodies in previous transplantation patients. Callender et al .[13] evaluated 650 patients awaiting kidney transplantation and found that HLA-DP-specific antibodies were more frequently present in patients with previous transplantation than in patients without prior transplantation (62% vs. 38%). Development of anti-DP antibodies associated with previous history of transplantation was also reported by Ling et al .[14], who investigated 1069 patients on a waiting list. They found that previous transplantation was significantly associated with the development of anti-DP antibodies (*p* = 0.002), while history of pregnancy or transfusion was not. Furthermore, several cases of preformed HLA-DP DSAs have been reported in the literature and all had history of previous transplantation [11, 12, 15]. Taken together, these observations all support the notion that history of previous transplantation is a risk factor for anti-DP antibody development. Therefore, HLA-DP typing and SAB analysis are recommended for re-transplant candidates to accurately identify HLA antibodies before transplantation.

Anti-DP antibodies in sera of sensitized patients are specific for epitopes shared by different HLA antigens. Previous studies described two sequence dimorphisms which accounted for immunodominant epitopes in HLA-DPB1: residue 56 (A or E) and residue 85-87 (GPM or EAV) [16]. The HLA-DPB1 alleles in our patient expressed A at residue 56 and antibodies reactive against 56E were produced after the first transplantation. Because the DPB1 allele in the second donor expressed 56E, the patient was re-sensitized toward 56E after the second transplantation (Table 2). Likewise, the DPA1 antibodies detected in the patient’s sera were reactive toward several DPA1 alleles that all shared the 50Q eplet, while the patient’s HLA-DPA1 alleles expressed the 50R eplet in the same region. These findings support the concept that anti-DP antibodies predominantly recognize broadly cross-reactive epitopes. Interestingly, both the patient and the second donor had DP2 antigens, but these were on different alleles. DPB1*02:02 in the patient expressed 56A, while DPB1*02:01 in the second donor expressed 56E. Therefore, it is prudent to consider HLA-DP epitope-based analysis for immunologic risk evaluation before transplantation in patients with HLA-DP antibodies.

**Table 2:** Amino acid sequence alignment of the donors and the recipient for DPA1 and DPB1

DPA1	Amino acid position								
	11	28	31	50-51	72-73	83	111	127	160
DPA1*02:02 (recipient)	M	E	Q	RA	TL	A	R	P	V
DPA1*01:03 (first and second donors)	A	E	M	QA	TL	T	K	L	F
**DPB1**	Amino acid position								
	8-11		33-36		55-57	65-69		76	84-87
DPB1*02:02 (recipient)	LFQG		EELV		EAE	ILEEE		M	GGPM
DPB1*05:01 (recipient)	LFQG		EELV		EAE	ILEEE		M	DEAV
DPB1*03:01 (first donor)	VYQL		EEFV		D**E**D	LLEEE		V	DEAV
DPB1*04:01 (first donor)	LFQG		EEFA		AAE	ILEEE		M	GGPM
DPB1*02:01 (second donor)	LFQG		EEFV		D**E**E	ILEEE		M	GGPM

The amino acid sequences were aligned according to their positions in the mature proteins

In summary, this case report illustrates the clinical importance of HLA-DPα and HLA-DPβ DSAs before kidney transplantation. Accuracy in determination of HLA antibodies before transplantation is critical for the transplant outcome. HLA-DP typing, SAB testing, and identification of epitope-specific antibodies would be beneficial for highly sensitized patients, and especially for patients with history of previous transplantation.

## Data Availability

The clinical data for this case are stored in our hospital medical records and cannot be shared.

## Abbreviations

AMR: Antibody-mediated rejection
ATG: Anti-thymocyte globulin
CDC-XM: Complement-dependent cytotoxicity crossmatch
DFPP: Double-filtration plasmapheresis
DSAs: Donor-specific antibodies
HLA: Human leukocyte antigen
MFI: Mean fluorescence intensity
PRA: Panel-reactive antibody
SAB: Single antigen bead
